# Ambulantes psychosoziales Screening in einem Hautkrebszentrum: Akzeptanz, psychosoziale Belastung und Inanspruchnahme von Beratungsangeboten: Eine Post-hoc-Analyse im Rahmen der Qualitätssicherung

**DOI:** 10.1007/s00105-024-05347-2

**Published:** 2024-05-08

**Authors:** Alexander Wünsch, Niklas Jeske, Natalie Röderer, Frank Meiss

**Affiliations:** 1 Psychoonkologischer Dienst, Medizinische Onkologie, Inselspital, Bern Universitätsspital, Universität Bern, Bern, Schweiz; 2 Psychosoziale Krebsberatungsstelle Freiburg, Tumorzentrum Freiburg – CCCF in Kooperation mit der Klinik für Psychosomatische Medizin und Psychotherapie, Universitätsklinikum Freiburg, Medizinische Fakultät, Albert-Ludwigs-Universität Freiburg, Hauptstr. 5a, 79104 Freiburg, Deutschland; 3 Klinik für Dermatologie und Venerologie, Universitätsklinikum Freiburg, Medizinische Fakultät, Albert-Ludwigs-Universität Freiburg, Hauptstr. 7, 79104 Freiburg, Deutschland

**Keywords:** Psychosozialer Distress, Ambulante psychosoziale Krebsberatung, Melanom, Subjektiver Betreuungswunsch, Screeninginstrumente, Psychosocial distress, Cancer counseling centers, Melanoma, Subjective desire for care, Screening instruments

## Abstract

**Hintergrund:**

Die psychosoziale Versorgung von Krebspatienten nimmt über den gesamten Verlauf der onkologischen Behandlung einen wichtigen Stellenwert ein. Seit 2015 ist das psychosoziale Screening in den Ambulanzen des Hauttumorzentrums in Freiburg implementiert. Wir präsentieren hier eine Post-hoc-Analyse im Rahmen der Qualitätssicherung.

**Fragestellung:**

Akzeptanz, psychosoziale Belastung und Inanspruchnahme wurden evaluiert. Explorativ untersuchten wir, welche Patienten- und Krankheitsmerkmale mit einer erhöhten subjektiven Belastung zusammenhängen.

**Material und Methoden:**

In einer Vollerhebung von 06/2015 bis 12/2015 wurden die Akzeptanz, die psychosoziale Belastung mittels Distress-Thermometer (DT) und der Beratungswunsch ausgewertet.

**Ergebnisse:**

Von 753 Patienten haben 345 (45,8 %) am psychosozialen Screening teilgenommen und Daten von 310 (m:w 174:136; 89,7 % Melanompatienten, mittlere Zeit seit Erstdiagnose 4,7 ± 3,9 Jahre) konnten ausgewertet werden. Die mittlere Belastung auf dem DT betrug 2,97 ± 2,83 (Median 2, Range 0 bis 10). Eine überschwellige Belastung (DT ≥ 5) wurde von 84 Patienten (28,8 %) angegeben; 34 Patienten (11 %) gaben einen Beratungswunsch an, und 23 Patienten nahmen das Beratungsangebot in Anspruch. Patienten mit überschwelliger Belastung waren jünger, befanden sich häufiger unter laufender oder kürzlich abgeschlossener medikamentöser Therapie und hatten häufiger einen Beratungswunsch.

**Diskussion:**

Neben der Erhebung der Belastung mit validierten Screeninginstrumenten stellt die Erhebung des subjektiven Betreuungswunsches einen wichtigen Parameter zur Identifikation von betreuungsbedürftigen Patienten dar. Junge Patienten und Patienten unter Systemtherapie sollten in den Fokus der Aufmerksamkeit rücken.

## Abstract (en)

**Background:**

Psychosocial care of cancer patients is an important aspect throughout the entire course of oncological treatment. Since 2015, psychosocial screening has been implemented in the outpatient clinics of the Skin Cancer Center in Freiburg. We present here a post hoc analysis in the context of a quality management program.

**Question:**

Acceptance, psychosocial distress and desire for counseling were evaluated. Exploratively, we investigated which patient and disease characteristics are related to increased subjective distress.

**Materials and methods:**

In a full survey from 06/2015 to 12/2015, we evaluated acceptance, psychosocial distress by distress thermometer (DT), and desire for counseling.

**Results:**

Of 753 patients, 345 (45.8%) participated in psychosocial screening and data from 310 (174 men, 136 women; 89.7% melanoma patients, mean time since initial diagnosis 4.7 ± 3.9 years) could be analyzed. The mean burden on the DT was 2.97 ± 2.83 (median 2, range 0–10). High distress (DT ≥ 5) was reported by 84 patients (28.8%). Thirty-four patients (11%) indicated a desire for counseling, and 23 patients took up the counseling offer. The patient group with high distress was younger, more often under ongoing or recently completed systemic therapy, and had more often a desire for counseling.

**Conclusion:**

In addition to assessing psychosocial distress with validated screening instruments, the survey of the subjective desire for care represents an important parameter for the identification of patients in need of care. Young patients and patients with ongoing systemic therapy should be the focus of attention.

Die psychosoziale Versorgung von Krebspatienten nimmt über den gesamten Verlauf der onkologischen Behandlung einen wichtigen und zunehmend wahrgenommenen Stellenwert ein. Im Nationalen Krebsplan (NKP) ist die angemessene psychoonkologische Betreuung ein fester Bestandteil einer umfänglichen onkologischen Betreuung von Krebspatienten in Deutschland. Im Handlungsfeld 2 des NKPs „Weiterentwicklung der onkologischen Versorgungsstrukturen und der Qualitätssicherung“ wird dies unter dem Ziel 9 mit der Formulierung „Alle Krebspatienten erhalten bei Bedarf eine angemessene psychoonkologische Versorgung“ herausgestellt [16].

## Hintergrund

In zertifizierten Hautkrebszentren (HKZ) in Deutschland wurden die wesentlichen strukturellen und personellen Voraussetzungen für eine psychoonkologische Versorgung von Melanompatienten bestätigt [27]. Der Versorgungsschwerpunkt lag hierbei im stationären Bereich [27]. Da entsprechend den Zertifizierungsvorgaben das psychosoziale Screening in allen Phasen der Erkrankung vorzuhalten und anzubieten ist [17], müssen Konzepte entwickelt werden, um auch die ambulant betreuten Patienten der Hauttumorzentren zu erreichen. Erhebungen aus deutschen Hauttumorzentren konnten zeigen, dass 30–47 % der ambulant betreuten Hautkrebspatienten (Schwerpunkt Melanompatienten) einen überschwelligen Distress angeben [7, 23, 24]. Bisher gibt es nur wenige Daten zu strukturierten psychosozialen Betreuungskonzepten im ambulanten Setting von Hautkrebszentren [7]. Des Weiteren ist bisher nicht erhoben, wie die Akzeptanz zur Teilnahme an einem psychosozialen Screening ist und in welchem Ausmaß niederschwellige Betreuungsangebote auf dem Boden eines strukturierten Screenings in Anspruch genommen werden. Seit 2015 erfolgen in den Ambulanzen des Hauttumorzentrums (HTZ) am Universitätsklinikum (UK) Freiburg das strukturierte psychosoziale Screening und die Betreuung von Hauttumorpatienten in Kooperation mit der psychosozialen Krebsberatungsstelle (KBS). Nach Etablierung des kooperativen Screeningkonzeptes erfolgte eine Post-hoc-Analyse im Rahmen der Qualitätssicherung. Im Rahmen der Analyse sollten die Akzeptanz, der Betreuungsbedarf und die Inanspruchnahme des strukturierten psychosozialen Betreuungskonzeptes in der ambulanten Versorgung von Hautkrebspatienten evaluiert werden.

## Fragestellungen

Ziel der Arbeit war es, im Rahmen des Qualitätssicherungsprojektes die folgenden Fragenstellungen zu untersuchen:

- Wie ist die Akzeptanz der Hautkrebspatienten, an einem freiwilligen niederschwelligen psychosozialen Screening teilzunehmen?
- Wie ausgeprägt sind psychosoziale Belastung, Beratungswunsch und Inanspruchnahme von Beratungsangeboten in der Patientengruppe?
- Welche Faktoren hängen in einer explorativen Analyse mit einer erhöhten psychosozialen Belastung bei Hautkrebspatienten zusammen?

## Studiendesign und Untersuchungsmethoden

Die vorliegenden Daten wurden in der Nachsorgesprechstunde des HTZ Freiburg und der KBS am Tumorzentrum Freiburg – CCCF (Comprehensive Cancer Center Freiburg) im Zeitraum vom 09.06.2015 bis 29.12.2015 erhoben. Dabei handelt es sich um Prozessdaten, die im Rahmen der klinischen Routine zur Identifikation der psychosozialen Belastungen und Feststellung des Beratungsbedarfes und -wunsches erfasst wurden und der Qualitätssicherung dienen sollen. Allen Patienten, die im Erhebungszeitraum einen Nachsorgetermin in der Tumornachsorgesprechstunde der Klinik für Dermatologie und Venerologie am UK Freiburg hatten, wurde ein psychosoziales Screening angeboten. Bei vorliegender Bereitschaft zur Teilnahme wurde der Screeningbogen ausgehändigt. Der Screeningbogen wurde während der Wartezeit auf den Untersuchungstermin von den Patienten ausgefüllt und den Mitarbeitern des Ambulanzteams zurückgegeben. Die Fragebögen wurden dann an die Mitarbeiter der KBS weitergeleitet. Neben einem einleitenden Informationstext beinhaltete der Fragebogen eine schriftliche Einwilligung zur Nutzung und Auswertung der Daten. Als Screeninginstrument wurden die deutsche Version des NCCN(National Comprehensive Cancer Network)-Distress-Thermometers (DT) sowie die Problemliste verwendet [15, 25]. Im DT werden die Patienten um eine Einschätzung ihrer psychosozialen Belastung in der letzten Woche auf einer visuellen Analogskala von 0 bis 10 gebeten (0 = gar nicht belastet; 10 = maximal belastet). Die Indikation für eine psychoonkologische Beratung ist bei einer überschwelligen Belastung ≥ 5 gegeben. Psychosoziale Belastung ist im DT ein bewusst breit gefasstes Konzept, das neben psychischen Belastungen wie Ängsten oder Traurigkeit auch sozioökonomische, berufliche oder familiäre Belastungen umfasst [26]. Die Problemliste fragt dementsprechend nach Vorliegen (ja/nein) von für onkologische Patienten typischen Problemen, eingeteilt in praktische Probleme, familiäre Probleme, emotionale Probleme, spirituelle/religiöse Belange, körperliche Probleme und sonstige Probleme [15, 26]. Darüber hinaus wurde der Patientenwunsch nach einer psychologischen oder sozialrechtlichen Beratung erfragt (ja/nein). Bei angegebenem Beratungswunsch und/oder einer überschwelligen Belastung wurden die Patienten von den Mitarbeitern der KBS kontaktiert, und eine Terminvereinbarung wurde angeboten. Beratungsschwerpunkte und die Häufigkeit der Beratungskontakte wurden dokumentiert.

Zusätzliche Variablen aus der klinischen Versorgung wurden für die Analysen herangezogen: bei allen Patienten Alter, Geschlecht und Jahre seit Erstdiagnose; bei Melanompatienten zusätzlich medikamentöse Therapie (keine/laufend/seit bis zu 1 Jahr abgeschlossen/seit über 1 Jahr abgeschlossen), Tumorstadium nach AJCC (American Joint Committee on Cancer), Rezidiv oder Progress (keines/innerhalb 1 Jahr/vor über 1 Jahr). Entsprechend den Vorgaben der Ethik-Kommission der Albert-Ludwigs-Universität Freiburg war ein Ethikvotum für diese Analyse im Rahmen der Qualitätssicherung und ohne Verwendung identifizierbarer Patientendaten nicht notwendig. Die schriftliche Zustimmung der Patienten zur Auswertung der Daten zu Zwecken der Qualitätssicherung erfolgte auf den Screeningbögen, Patienten ohne dokumentierte Einwilligung wurden von der Auswertung ausgeschlossen. Die Datenerhebung und Auswertung erfolgte unter Einhaltung der Deklaration von Helsinki in ihrer aktuellen Fassung.

## Auswertung

### Deskriptive Analyse

Die Auswertung der Daten erfolgte mit IBM SPSS Statistics (Version 29; IBM Corp, Armonk, NY, USA). Zunächst wurden Häufigkeit und prozentualer Anteil von

- am Screening teilnehmenden Patienten,
- Patienten mit einer überschwelligen Belastung im Distress-Thermometer (Werte ≥ 5) sowie
- von Patienten mit dem Wunsch nach psychologischer oder Sozialberatung

bestimmt. Die darauf basierende Inanspruchnahme der Beratung wurde ebenfalls erhoben. Wir bestimmten zusätzlich den durchschnittlichen Distresswert mit den zugehörigen Streuungsmaßen. Ebenso deskriptiv ausgewertet wurden die soziodemografischen Patientenvariablen Alter und Geschlecht sowie die krankheitsbezogenen Eigenschaften.

### Gruppenvergleiche

Anschließend untersuchten wir, inwiefern sich die Patientengruppen mit überschwelligem Distress (Werte ≥ 5) und geringem Distress (Werte < 5) hinsichtlich ihrer soziodemografischen und krankheitsbezogenen Eigenschaften unterscheiden. Dazu verwendeten wir bei kategorialen Variablen (z. B. Geschlecht oder Tumorstadium) den Exakten Fisher-Test und den Chi-Quadrat-Test, bei Alter und Zeit seit Erstdiagnose einen t‑Test für unabhängige Stichproben. Das Signifikanzniveau wurde auf *p* < 0,05 festgelegt.

### Multiple Regressionsanalyse

Um mögliche Zusammenhänge über das gesamte Spektrum der Distresswerte (0 bis 10) zu untersuchen, führten wir zusätzlich eine explorative multiple Regressionsanalyse mit der abhängigen Variable Distresswert und den oben beschriebenen Prädiktorvariablen durch. Da das Tumorstadium nach AJCC und Informationen zum Auftreten eines Rezidivs nur für Melanompatienten vorlagen, wurden die Patienten mit anderen Hautmalignomen (*n* = 29) in dieser Analyse ausgeschlossen. Alle Prädiktorvariablen wurden in die Analyse einbezogen (Einschluss).

## Ergebnisse

### Stichprobe

**Abb. 1:**
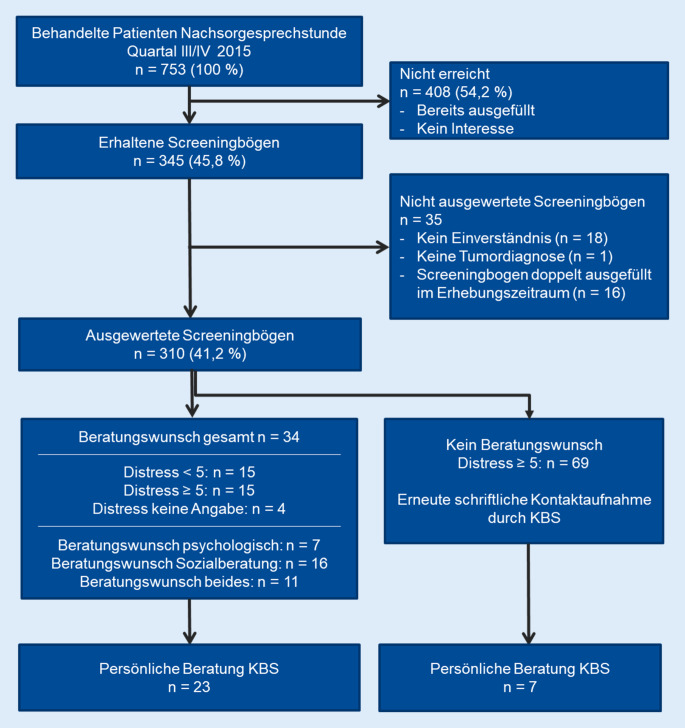
Flussdiagramm zur Stichprobe. *KBS* Krebsberatungsstelle

Im Erhebungszeitraum von 6 Monaten wurden 753 Patienten in der Nachsorgesprechstunde des Hauttumorzentrums behandelt und hatten im Durchschnitt 1,35 Nachsorgekontakte (Gesamtkontakte 1016, Range 1 bis 4 Kontakte pro Patient). Von diesen 753 Patienten haben 345 (45,8 %) am psychosozialen Screening teilgenommen und die Screeningbögen ausgefüllt; 94,8 % der Patienten (*n* = 326) hatten ihr Einverständnis zur Auswertung der Daten gegeben. Von den 326 Patienten hatten 16 Patienten im Erhebungszeitraum den Fragebogen zweimalig ausgefüllt. Hiervon wurde jeweils der Fragebogen zum früheren Zeitpunkt für die Auswertung verwendet. Insgesamt wurden 310 Patienten in die Auswertung eingeschlossen. Bei allen Patienten mit 2 vorliegenden Fragebögen wurden die Fragebögen auf unterschiedliche Werte geprüft. Sie unterschieden sich jedoch nicht. Zum ersten Zeitpunkt war der erhobene Distress im Mittelwert (MW) 2,8 (Median 2) und zum zweiten Zeitpunkt MW 3,0 (Median 2). Alle Patienten gaben keinen Beratungswunsch zu den verschiedenen Zeitpunkten an. Das Flussdiagramm zur Patientenrekrutierung ist in Abb. 1 dargestellt.

### Merkmale der Untersuchungsstichprobe

In der Gesamtgruppe befanden sich 136 Frauen (43,9 %) und 174 Männer (56,1 %). Der Großteil der Patienten (*n* = 278; 89,7 %) befand sich in der Nachsorgesprechstunde mit der Diagnose eines malignen Melanoms, deutlich geringer war der Anteil von Patienten mit Hautlymphomen (*n* = 22, 7,1 %), Merkel-Zell-Karzinom (*n* = 5, 1,6 %), Plattenepithelkarzinom (*n* = 3, 1,0 %) und weiteren selten Hautmalignomen (*n* = 2, 0,6 %) (Tab. 1). In der Gruppe der Melanompatienten befanden sich 119 im Stadium I (42,8 %), 80 im Stadium II (28,8 %), 71 im Stadium III (25,5 %) und nur 8 Patienten im Stadium IV (2,9 %) (entsprechend der 2015 gültigen Version 7 der AJCC-Klassifikation von 2009 [3]). Die mittlere Zeit seit Erstdiagnose betrug 4,7 ± 3,9 Jahre (Median 3,8, Range 0 bis 19,8 Jahre). Nur 6,8 % der Patienten (*n* = 19) befanden sich zum Zeitpunkt ihres psychosozialen Screenings unter einer laufenden systemischen medikamentösen Therapie, 5 % der Patienten (*n* = 14) hatten ihre medikamentöse Therapie innerhalb des letzten Jahres abgeschlossen, 34,9 % der Patienten über 1 Jahr vor dem Zeitpunkt des Screenings. Bei 6,1 % der Stichprobe (*n* = 17) war es innerhalb von 1 Jahr vor dem Screening zum Auftreten eines Rezidivs oder eines Tumorprogresses gekommen (Tab. 1).

**Tab. 1:** Patientencharakteristika der Gesamtgruppe (*n* = 310)

	Gesamt (*n* = 310)		Frauen (*n* = 136, 43,9 %)		Männer (*n* = 174, 56,1 %)	
**Patientenvariablen**						
*Alter*						
Mittelwert (SD)	61,7 (14,9)		59,8 (16,0)		63,2 (13,8)	
Median (IQR)	63 (22)		63 (22,5)		64 (20,8)	
Range	22–91		22–91		24–90	
*Altersgruppen*	*n*	*%*	*N*	*%*	*n*	*%*
22–49	66	21,3	37	27,2	29	16,7
50–65	102	32,9	40	29,4	62	35,6
66–91	142	45,8	59	43,4	83	47,7
**Krankheitsvariablen**						
*Tumorentität*						
Melanom	278	89,7	128	94,1	150	86,2
Kutane Lymphome	22	7,1	5	3,6	17	9,8
Merkel-Zell-Karzinom	5	1,6	1	0,7	4	2,3
Plattenepithelkarzinom	3	1,0	1	0,7	2	1,1
Weitere Hautkrebsarten^a^	2	0,6	1	0,7	1	0,6
*Jahre seit Erstdiagnose*						
Mittelwert (SD)	4,7 (3,9)		4,4 (3,4)		4,9 (4,2)	
Median (IQR)	3,8 (5,3)		3,6 (4,9)		3,9 (5,6)	
Range	0–19,8		0–17,0		0,1–19,8	
*Tumorstadium*^*b,c*^	*n*	*%*	*N*	*%*	*n*	*%*
Stadium I	119	42,8	63	49,2	56	37,3
Stadium II	80	28,8	31	24,2	49	32,7
Stadium III	71	25,5	32	25,0	39	26,0
Stadium IV	8	2,9	2	1,6	6	4,0
*Medikamentöse Therapie*^*c*^						
Keine	148	53,2	75	58,6	73	48,7
Laufend	19	6,8	5	3,9	14	9,3
Abgeschlossen < 1 Jahr	14	5,0	7	5,5	7	4,7
Abgeschlossen > 1 Jahr	97	34,9	41	32,0	56	37,3
*Rezidiv/Progress*^*c*^						
Kein Rezidiv	229	82,4	109	85,2	120	80,0
Vor < 1 Jahr	17	6,1	6	4,7	11	7,3
Vor > 1 Jahr	32	11,5	13	10,2	19	12,7

*n* Anzahl, *SD* Standardabweichung, *IQR* Interquartilsabstand

^a^ Adnextumoren und kutane Sarkome

^b^ 7. Version AJCC-Klassifikation 2009

^c^ Nur Melanompatienten

### Distress-Thermometer: durchschnittliche Belastung

Von den 310 Patienten hatten 292 (94,2 %) vollständige Angaben für das Distress-Thermometer (DT) gemacht. Die mittlere Belastung auf dem DT betrug 2,97 ± 2,83 (Median 2, Interquartilsabstand 5, Range 0–10). Eine überschwellige Belastung (DT ≥ 5) wurde von 84 Patienten (28,8 %) angegeben (Tab. 2).

**Tab. 2:** Patientencharakteristika der gering und überschwellig belasteten Patientengruppe (*n* = 292)

	DT-Wert < 5 (*n* = 208, 71,2 %)		DT-Wert ≥ 5 (*n* = 84, 28,8 %)		*p*
**Patientenvariablen**					
*Alter*					
Mittelwert (SD)	62,5 (14,2)		58,2 (15,6)		*0,023*^c^
Median (IQR)	65 (22)		61 (22,8)		
Range	24–90		22–87		
*Altersgruppen*	*n*	*%*	*n*	*%*	*0,021*^*d*^
22–49	39	18,8	24	28,6	
50–65	66	31,7	33	39,3	
66–90	103	49,5	27	32,1	
*Geschlecht*					0,358^e^
Weiblich	84	40,4	40	47,6	
Männlich	124	59,6	44	52,4	
**Beratungswunsch**					
Gesamt	15	7,2	15	17,8	**0,007**^d^
Psychologisch	7	1,5	10	4,7	**0,010**^e^
Sozialberatung	12	3,8	11	6,0	0,052^e^
Beides	4	1,9	6	7,1	**0,036**^e^
**Krankheitsvariablen**					
*Jahre seit Erstdiagnose*					
Mittelwert (SD)	4,8 (3,8)		4,5 (4,0)		0,452^c^
Median (IQR)	3,98 (5,5)		3,25 (5,1)		
Range	0–19,2		0,3–19,8		
*Tumorstadium*^*a,b*^	*n*	*%*	*n*	*%*	*0,822*^*d*^
Stadium I	85	44,0	26	37,1	
Stadium II	52	28,0	22	31,4	
Stadium III	50	25,9	20	28,6	
Stadium IV	6	3,1	2	2,9	
*Medikamentöse Therapie*^*a*^					**0,005**^**d**^
Keine	109	56,5	30	42,9	
Laufend	10	5,2	8	11,4	
Abgeschlossen < 1 Jahr	5	2,6	8	11,4	
Abgeschlossen > 1 Jahr	69	35,8	24	34,3	
*Rezidiv/Progress*^*a*^					0,373^d^
Kein Rezidiv	160	82,9	55	78,6	
Vor < 1 Jahr	10	5,2	7	10,0	
Vor > 1 Jahr	23	11,9	8	11,4	

*n* = Anzahl, *DT* Distress-Thermometer, *SD* Standardabweichung, *IQR* Interquartilsabstand, *p in Fettdruck* signifikanter Unterschied

^a^ Nur Melanompatienten, *n* = 263, DT < 5: *n* = 193, DT ≥ 5: *n* = 70

^b^ 7. Version AJCC-Klassifikation 2009

^c^ t‑Test für unabhängige Stichproben

^d^ Chi-Quadrat-Test

^e^ Exakter Fisher-Test

### Unterstützungsbedürfnisse und Inanspruchnahme der psychologischen Beratung und Sozialberatung

Unabhängig ihres Distresswerts gaben 34 der 310 Patienten (11 %) einen Beratungswunsch an, davon 7 Patienten für die psychologische Beratung, 16 Patienten für die Sozialberatung und 11 Patienten für beide Beratungen. Von diesen 34 Personen wurden nach dem Screening mit 23 Personen Beratungen (68 %) durchgeführt. Weitere 69 Personen mit einem DT ≥ 5, aber ohne Beratungswunsch wurden schriftlich kontaktiert und erneut auf das Beratungsangebot hingewiesen und 7 Personen (10 %) im weiteren Verlauf beraten (Abb. 1).

### Unterschiede zwischen gering und überschwellig belasteten Patienten

Im DT gaben 84 Patienten (28,8 %) eine überschwellige Belastung mit einem DT ≥ 5 und 208 Patienten (71,2 %) eine geringe Belastung mit einem DT < 5 an (Tab. 2).

#### *Patientenvariablen*.

In der Gruppe der gering belasteten Patienten lag das Durchschnittsalter um etwa 4 Jahre höher als in der Gruppe der belasteten Patienten (62,5 Jahre vs. 58,2 Jahre, t‑Test, *t*(290) = 2,29, *p* = 0,023). Insbesondere der Anteil der unter 65-Jährigen unterschied sich zwischen der unbelasteten Gruppe (50,5 %) und der belasteten Gruppe (67,9 %) (Chi-Quadrat-Test, χ^2^(2) = 7,74, *p* *=* 0,021). Das Geschlechterverhältnis unterschied sich nicht statistisch signifikant zwischen den beiden Gruppen.

#### Beratungswunsch.

In beiden Gruppen hatten jeweils 15 Patienten mindestens einen Beratungswunsch (psychologische Beratung, Sozialberatung oder beides). Das entspricht in der gering belasteten Gruppe einem Anteil von 7,2 %, in der überschwellig belasteten Gruppe einem Anteil von 17,8 % (Chi-Quadrat-Test, χ^2^(1) = 7,36, *p* = 0,007). Aufgeschlüsselt nach den unterschiedlichen Beratungswünschen bestanden statistisch signifikante Unterschiede beim Wunsch nach psychologischer Beratung (4,7 % vs. 1,5 %, *p* = 0,010, Exakter Fisher-Test) und beim Wunsch nach beiden Beratungen (7,1 % vs. 1,9 %, Exakter Fisher-Test *p* *=* 0,038), jedoch nicht für den Wunsch nach Sozialberatung.

#### Krankheitsbezogene Eigenschaften.

Bei den krankheitsbezogenen Eigenschaften zeigte sich ein statistisch signifikanter Unterschied hinsichtlich der medikamentösen Therapie (Chi-Quadrat-Test, χ^2^(3) = 12,88, *p* = 0,005) mit anteilig mehr Patienten unter laufender oder im letzten Jahr abgeschlossener medikamentöser Therapie in der überschwellig belasteten Gruppe.

### Zusammenhänge zwischen Patienteneigenschaften und Distresswert von 0 bis 10

Das Modell unserer explorativen Regressionsanalyse (Tab. 3) mit den aufgenommenen Prädiktorvariablen unterschied sich signifikant vom Nullmodell (*R*^2^ = 0,13, Modell χ^2^(8,2 53) = 4,53, *p* < 0,001). Statistisch signifikante Zusammenhänge mit dem Distresswert zeigten sich für den Wunsch nach psychologischer Beratung (*p* = 0,010) und für den Wunsch nach Sozialberatung (*p* = 0,004), jedoch nicht für die Patientenvariablen oder Krankheitsvariablen.

**Tab. 3:** Multiple Regression für Distresswert (0 bis 10) bei Melanompatienten (*n* = 263)

	*b*	Unteres 95 %-KI für *b*	Oberes 95 %-KI für *b*	*p*
*Patientenvariablen*				
Alter	−0,02	−0,04	0,00	0,111
Geschlecht	−0,10	−0,78	0,59	0,780
*Beratungswunsch*				
Psychologisch	2,06	0,50	3,62	**0,010**
Sozialberatung	2,08	0,68	3,47	**0,004**
*Krankheitsvariablen*				
Jahre seit Erstdiagnose	−0,03	−0,14	0,07	0,538
Tumorstadium	0,01	−0,42	0,43	0,973
Medikamentöse Therapie	0,14	−0,15	0,43	0,343
Rezidiv/Progress	0,06	−0,49	0,60	0,834

*KI* Konfidenzintervall, *b* Regressionskoeffizient, *p in Fettdruck* signifikanter Unterschied

## Diskussion

In der vorliegenden Studie wurden Daten von 310 Patienten aus der Tumornachsorgesprechstunde des zertifizierten Hauttumorzentrums am Uniklinikum Freiburg ausgewertet, denen im Rahmen der Routineversorgung ein strukturiertes psychosoziales Screening in Kooperation mit einer dem Universitätsklinikum angeschlossenen KBS angeboten wurde. Über den Zeitraum eines halben Jahres wurden anhand der vollständigen Erhebung der Patientenkohorte die Akzeptanz, der Betreuungsbedarf und die Inanspruchnahme des strukturierten psychosozialen Betreuungskonzeptes in der ambulanten Versorgung von Hautkrebspatienten evaluiert.

### Akzeptanz.

Die Daten wurden im Routinebetrieb in der ambulanten Tumornachsorgesprechstunde erhoben. Dabei wurde in einem halben Jahr eine Vollerhebung der Patienten angestrebt. Es waren 45,8 % der Patienten (345 von 735 Patienten der Nachsorgesprechstunde) bereit, am Screening teilzunehmen. Die häufigsten Gründe für eine Nichtteilnahme waren fehlendes Interesse oder bereits erfolgte Teilnahme am Screening bei vorausgegangenen Ambulanzkontakten, eine detaillierte Non-Responder-Analyse wurde jedoch im Rahmen unserer Erhebung nicht durchgeführt. Die Ablehnungsquote entspricht jedoch der aus zahlreichen anderen Studien [25, 29, 32, 33], in denen als häufigster Ablehnungsgrund ein fehlendes Interesse zum Zeitpunkt der Befragung angegeben wurde. Responder/Non-Responder-Analysen zeigen, dass Screeningteilnehmer jünger sind und eine bessere Schulbildung haben [20, 25, 29]. Die Teilnahmequote in unserer Analyse mit fast 50 % außerhalb eines Studiensettings ist akzeptabel. Eine Stichprobenverzerrung durch den Anteil von Non-Respondern erscheint unwahrscheinlich, da unsere Ergebnisse eine hohe Übereinstimmung mit den Daten aus anderen Arbeiten zeigen, in denen die Non-Responder-Quote deutlich geringer war [4, 23, 24]. Eine höhere Akzeptanz könnte durch aktivere Einbindung ärztlicher Mitarbeiter [6, 12] und von onkologischen Fachpflegekräften [10] in die Screeningabläufe erzielt werden. Zudem konnte durch den Einsatz von E‑Health-Tools die Akzeptanz von Krebspatienten, an strukturierten Nachsorgeprogrammen (inklusive des psychosozialen Screenings) teilzunehmen, erhöht werden [11]. In den Zertifizierungsvorgaben anderer organspezifischer Zentren (z. B. Kopf-Hals-Tumorzentrum, Lungenkrebszentrum) wurde zwischenzeitlich die Kennzahl der psychoonkologischen Betreuungsquote durch eine psychoonkologische Screeningquote mit ≥ 65 % ersetzt [19]. Es ist davon auszugehen, dass diese Kennzahl auch in die Zertifizierungskriterien der Hautkrebszentren Einzug finden wird. Vor diesem Hintergrund erscheint es sinnvoll, bereits jetzt die strukturellen Voraussetzungen hierfür zu etablieren.

### Belastung und Beratungsbedarf.

Gut ein Drittel (28,8 %) der befragten Patienten gab eine überschwellige Belastung von 5 oder mehr auf dem DT an als wichtigen Surrogatwert für einen Betreuungsbedarf [15, 26]. Der Anteil der überschwellig belasteten Patienten in unserer Kohorte liegt somit etwas unter den Werten vergleichbarer Erhebungen aus anderen zertifizierten Hauttumorzentren, in denen bei 30–47 % der Befragten ein erhöhter Distress erfasst wurde [5–7, 23, 24]. Entsprechend ist die gemessene mittlere Belastung unserer Kohorte mit 2,97 ± 2,83 (Median 2) geringer im Vergleich zu den Erhebungen von Mayer et al. (4,3 ± 2,64) [24] und Loquai et al. (3,9 ± 3,0) [23]. Diese Unterschiede könnten zumindest teilweise durch das lange Zeitintervall seit Erstdiagnose (im Mittel 4,7 Jahren) sowie durch die geringen Anteile von Patienten unter laufender Systemtherapie (6,8 %) und Patienten im Stadium IV (2,9 %) in unserer Erhebung begründet sein. So wurden höhere Belastungswerte bei Melanompatienten mit kürzerem Zeitintervall seit Erstdiagnose [4, 24], laufender medikamentöser Tumortherapie [23, 24] und höherem Tumorstadium [4, 23, 24] berichtet. Es gaben 11 % in der Patientenkohorte mindestens einen Beratungswunsch an. In der gering belasteten Gruppe lag der Anteil bei 7,2 %, in der überschwellig belasteten Gruppe mit 17,8 % deutlich höher. Dennoch äußert der Großteil der überschwellig belasteten Patienten keinen Beratungswunsch. Dies deckt sich mit früheren Studienergebnissen, in denen 50–70 % der nachweislich belasteten Patienten ein Beratungsangebot ablehnten [9, 29]. Die meist genannten Gründe waren, dass sie mit ihrer Belastung lieber alleine umgehen möchten, die subjektiv wahrgenommene Belastung nicht hoch genug sei oder dass andere Ressourcen zur Unterstützung genutzt werden [9, 29]. Im Regressionsmodel war lediglich der Patientenwunsch nach psychologischer und Sozialberatung signifikant, sonst aber keiner der anderen untersuchten Prädiktoren. Dies unterstreicht die Bedeutung der Erhebung des Beratungswunsches im Screeningprozess, und unsere Daten werden unterstützt durch die Ergebnisse anderer Studien, die die Bedeutung des Patientenwunsches als wichtigen Parameter herausgestellt haben [24, 29]. Zudem zeigt sich, dass der angegebene Distresswert bei einem Teil der Patienten einen bestehenden Beratungswunsch nicht zuverlässig erfassen kann. In der Studie von Pichler et al. [30] konnte gezeigt werden, wie wichtig es ist, in der Psychoonkologie sowohl auf die aktuellen klinischen Merkmale (z. B. krankheitsbezogene Eigenschaften) als auch auf die dauerhaften Patientenvariablen einzugehen. Diese Informationen könnte man nutzen, um das psychosoziale Beratungskonzept gezielter einzusetzen.

Alle Patienten mit Beratungswunsch wurden aktiv von den Mitarbeitenden der KBS kontaktiert, und 68 % dieser Patienten nahmen eine Beratung in der KBS in Anspruch. Die Gründe für die Nichtinanspruchnahme der Beratung trotz angegebenen Beratungswunsches und Kontaktaufnahme wurden nicht erhoben. Zehn Prozent der Patienten mit überschwelligem Distresswert und ohne angegebenen Beratungswunsch konnten durch eine postalische Kontaktaufnahme mit erneutem Hinweis auf die Betreuungsangebote für eine Beratung in der KBS erreicht werden. Insgesamt erscheint die erreichte Betreuungsquote gering, entspricht aber den Erfahrungen aus anderen psychosozialen Krebsberatungsstellen in Deutschland, in den Hautkrebspatienten im Vergleich zu Patienten mit anderen Krebsentitäten nur in geringerem Ausmaß die Betreuungsangebote in Anspruch nehmen [21]. Dieser Besonderheit in der psychosozialen Unterstützung von Melanompatienten wird auch in der aktuellen S3-Leitlinie Psychoonkologie Rechnung getragen, dahingehend, dass ein geringerer Personalschlüssel für Psychoonkologen vorgehalten werden muss [18].

### Unterschied der gering und stark belasteten Patientengruppe.

Umso wichtiger scheint es für diesen geringeren Anteil an belasteten Patienten mit Betreuungsbedarf bzw. -wunsch zu sein, das Angebot und den Zugang zum Angebot zu optimieren. Entsprechend unseren Daten sind insbesondere Patienten unter laufender und kürzlich abgeschlossener medikamentöser Tumortherapie sowie jüngere Patienten in den Fokus zu nehmen. Da in zahlreichen Hautkrebszentren Patienten unter Systemtherapie in speziellen Systemtherapieeinheiten behandelt werden, könnten hier die personellen und strukturellen Voraussetzungen für eine intensivere psychosoziale Betreuung geschaffen werden. Die Umsetzung eines Stepped-Care-Betreuungskonzeptes stellt ein Modell für eine optimierte psychosoziale Versorgung dar, in dem bedarfsorientiert Betreuungsquoten von bis zu 50 % erreicht wurden [1, 28].

## Limitationen

Die Idee für diese Publikation entstand im Rahmen eines Qualitätssicherungsprojektes eines gemeinsam eingeführten strukturierten psychosozialen Screeningverfahrens in den Nachsorgesprechstunden des Hauttumorzentrums in Freiburg. Die Studie beschreibt und analysiert Post-hoc-Daten mit dem Vorteil einer hohen ökologischen Validität. Nachteile bestehen jedoch darin, dass nicht alle Faktoren systematisch erfasst wurden und teilweise nur Daten für die erfassten Melanompatienten ausgewertet werden konnten. Ein weiterer Nachteil besteht in der monozentrischen Erhebung. Eine multizentrische Studie im longitudinalen Design kann validere Daten erheben. Gleichwohl sei darauf hingewiesen, dass unsere Ergebnisse sich mit anderen Studien decken [23, 24, 33]. Die Integration der psychoonkologischen Versorgung in Tumorzentrum ist in Fachkreisen unumstritten [18]. Screeningverfahren erscheinen hierbei unter Berücksichtigung von verfügbarer Zeit und Ressourcen die beste Möglichkeit, belastete Patienten systematisch zu identifizieren und einem psychoonkologischen Unterstützungsangebot zuzuführen. Dennoch nimmt auch in unserer Erhebung ein beachtlicher Teil hochbelasteter Patienten keine psychoonkologische Unterstützung in Anspruch [8, 9, 29]. Die hohe Bedeutung des individuellen Beratungswunsches wird auch in unserer Studie deutlich, ob die alleinige Erfassung des Beratungswunsches als Screeningbestandteil ausreichend ist, kann anhand unserer Daten nicht abschließend beurteilt werden.

Eine weitere Limitation unserer Studie liegt darin, dass sich das Therapieangebot für Hautkrebspatienten seit dem Erhebungszeitpunkt in 2015 durch die Etablierung der neuen medikamentösen Therapieoptionen (Immuntherapie und zielgerichtete Therapie) maßgeblich verändert hat. Hierdurch wird sich der Anteil an Langzeitüberlebenden (Cancer Survivers) [31], Patienten in höheren Tumorstadien (Stadium III/IV) sowie Patienten unter laufender Tumortherapie in den Nachsorgeeinrichtungen an Hauttumorzentren deutlich erhöhen [2] und ist in der vorliegenden Arbeit aus dem Jahr 2015 nicht abgebildet. Dies könnte die durchschnittliche psychosoziale Belastung und den Beratungsbedarf der in den Nachsorgesprechstunden betreuten Patienten verändern [2, 13, 22]. Ein aktuelles Review dokumentiert eine hohe Prävalenz von psychologischen Belastungen, praktischen und finanziellen Problemen bei mit Checkpointinhibitoren behandelten Patienten [14], sodass diese Patientengruppen besonders von strukturierten psychoonkologischen Screening- und Beratungsangeboten profitieren werden [13]. Wir können über unsere Studie Erkenntnisse aus dem beschriebenen Erhebungszeitraum darstellen und regen an, die beschriebenen Themen immer wieder zu prüfen und aufzugreifen.

## Fazit für die Praxis

- Das am Hauttumorzentrum Freiburg etablierte und mittlerweile verstetigte Konzept eines psychosozialen Screeningkonzeptes in Kooperation mit der Psychosozialen Krebsberatungsstelle stellt eine Option zur Umsetzung der Zertifizierungsvorgaben dar.
- Neben dem Einsatz validierter Screeninginstrumente sollte zudem explizit der individuelle Beratungswunsch erfasst werden.
- Besonders belastete Patientengruppen (jüngere Patienten, Patienten unter laufender Systemtherapie) sollten in den Blick der Aufmerksamkeit rücken.
- Aufgrund der Weiterentwicklung der medikamentösen Therapieoptionen ist mit einer Zunahme von Langzeitüberlebenden, Patienten in höheren Tumorstadien und Patienten unter laufender Therapie auszugehen. Der Bedarf an psychosozialer Unterstützung dieser Patientengruppen scheint hoch zu sein und muss für die zukünftige Ausrichtung des psychosozialen Screenings und der Betreuungsangebote berücksichtigt werden.
